# A Distributed Oscillatory Network Model of Parkinsonian Tremor: Integrating Basal Ganglia and Cerebello-Thalamic Circuits

**DOI:** 10.5334/tohm.1198

**Published:** 2026-06-15

**Authors:** Victor Fellipe Bispo Macêdo, Alana Madeiro de Melo Barboza, Yasmin Lopes Silva Nogueira, Amanda Sena Cocivera Machado, Nilcele Freire de Oliveira

**Affiliations:** 1 Universidade Federal de Alagoas, UFAL, Maceió, Alagoas, Brazil; 2 Universidade de Maceió, UNIMA, Maceió, Alagoas, Brazil; 3 Centro Universitário de Maceió, CESMAC, Maceió, Alagoas, Brazil

**Keywords:** Parkinsonian tremor, Neural oscillations, Network model, Cerebellum, Basal ganglia

## Abstract

**Background:**

Parkinsonian tremor remains one of the least understood motor manifestations of Parkinson’s disease. Unlike bradykinesia and rigidity, tremor often shows weak correlation with dopaminergic degeneration and variable responses to pharmacological and surgical treatments, suggesting mechanisms beyond classical basal ganglia dysfunction. Increasing evidence indicates that tremor may arise from interactions between multiple motor circuits rather than from a single oscillatory generator.

**Methods:**

This mechanistic review integrates evidence from electrophysiological recordings, neuroimaging studies, lesion observations, and computational modeling studies investigating the neural mechanisms of parkinsonian tremor. Relevant literature addressing basal ganglia circuits, cerebello–thalamo–cortical pathways, and tremor-related neural oscillations was analyzed to develop a conceptual network framework.

**Results:**

Available evidence supports the involvement of a distributed oscillatory network in tremor generation. Within this framework, basal ganglia circuits, particularly the subthalamic nucleus–globus pallidus externa loop, act as primary oscillatory generators. Cerebello–thalamo–cortical pathways and brainstem nuclei modulate synchronization and amplitude of tremor-related activity, while thalamic nuclei function as resonance hubs integrating network oscillations and translating them into rhythmic motor output.

**Discussion:**

Conceptualizing parkinsonian tremor as a distributed network disorder helps explain several clinical observations, including the dissociation between tremor and other motor symptoms, variability in levodopa responsiveness, and heterogeneous tremor phenotypes. This framework also highlights the potential relevance of oscillatory biomarkers and network-based neuromodulation strategies, including adaptive deep brain stimulation, for future therapeutic approaches.

## Introduction

Parkinson’s disease (PD) is the second most common neurodegenerative disorder worldwide and one of the most prevalent conditions within this group [1,2,3,4]. It is primarily characterized by the progressive loss of dopaminergic neurons [5], leading to altered neural activity across the basal ganglia (BG), thalamus (TH), and cortical motor systems [6]. The disease imposes a substantial functional, social, and economic burden, significantly impairing daily living and quality of life.

Among the cardinal motor symptoms, bradykinesia, rigidity and rest tremor [7,8,9], tremor is observed in approximately 70–100% of patients [10] and typically ranges between 3–9 Hz [6,11]. Unlike other motor features, tremor remains the least well-understood manifestation of PD and is not fully explained by classical nigrostriatal dopaminergic dysfunction [6,12,13]. Its variable response to levodopa, often less robust than that observed for bradykinesia and rigidity [12,14,15], supports the coexistence of dopamine-dependent and dopamine-independent mechanisms.

Existing models do not fully account for several key features of parkinsonian tremor, including its dissociation from other motor symptoms, its variable response to dopaminergic therapy, and the heterogeneity of clinical phenotypes. In this context, we present a conceptual mechanistic review integrating experimental, neurophysiological, imaging, and computational evidence to propose an updated framework for tremor generation in PD.

**Figure 1:**
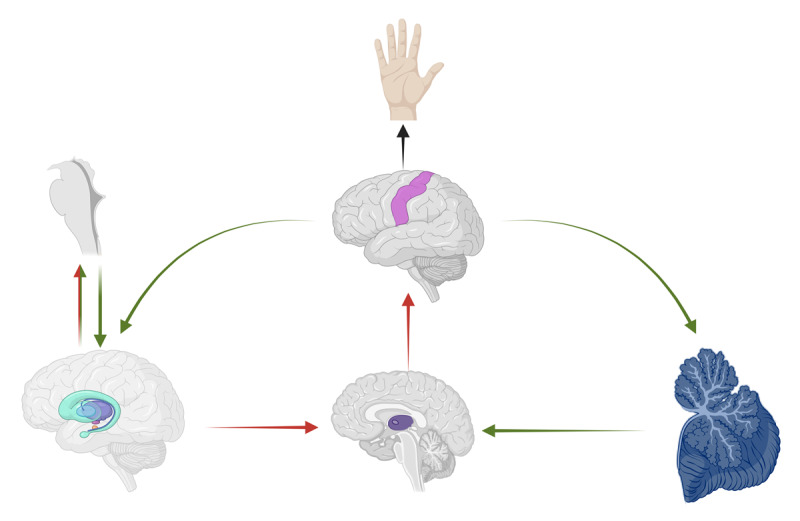
**Distributed oscillatory network underlying parkinsonian tremor**. Schematic representation of the proposed network model in which parkinsonian tremor emerges from dynamic interactions among basal ganglia, thalamic, cerebellar, and cortical motor circuits. Oscillatory activity generated within basal ganglia loops (left) is transmitted to the thalamus (center), where it is integrated and relayed to the sensorimotor cortex (top), ultimately leading to peripheral tremor expression. The cerebellum (right) contributes to modulation and synchronization of network activity through cerebello–thalamo–cortical pathways. Bidirectional connections between these structures (arrows) reflect continuous feedback and state-dependent modulation within the motor network.

In this review, we propose that parkinsonian tremor emerges from dynamic interactions between basal ganglia oscillatory generators, cerebellar modulatory circuits, and thalamocortical resonance mechanisms (Figure 1).

## Methods

This study was designed as a structured narrative review aimed at integrating mechanistic evidence across different experimental and clinical domains. Between November 2025 and January 2026, PubMed and Scopus were searched using “Parkinson’s disease”, “rest tremor”, “subthalamic nucleus”, “thalamus” and “neuronal oscillation”. The search was limited to articles published in English, without a temporal restrictions. This strategy yielded 141 articles in PubMed and 358 articles in Scopus.

Eligible studies included original research investigating mechanisms relevant to the pathophysiology of parkinsonian tremor. Evidence from human electrophysiology, neuroimaging, lesion studies, and computational models was integrated. When available, priority was given to human data, followed by converging evidence across experimental modalities. Studies were screened based on title and abstract, followed by full-text assessment when appropriate.

**Figure 2:**
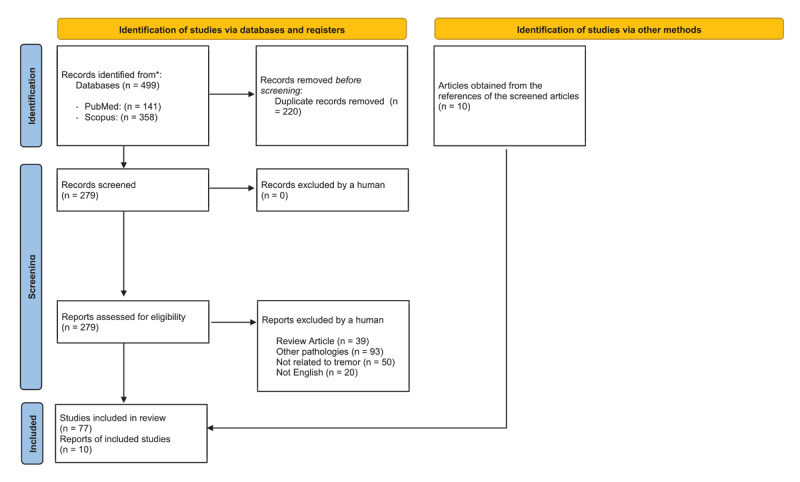
**Flow diagram summarizing the steps involved in the literature search**. Flowchart summarizing the identification, screening, eligibility, and inclusion of studies in the structured narrative review. A total of 499 records were identified through database searches (PubMed: n = 141; Scopus: n = 358), with 220 duplicates removed prior to screening. After screening 279 records, no studies were excluded at this stage. All 279 reports were assessed for eligibility, of which 202 were excluded (review articles: n = 39; other pathologies: n = 93; not related to tremor: n = 50; non-English: n = 20). An additional 10 studies were identified through reference screening. In total, 77 studies were included in the review.

Additional references were identified through manual searches of cited literature, yielding 10 studies. After excluding studies not relevant to this review, duplicates, and those published in languages other than English, a total of 90 articles were included (Figure 2). In cases of conflicting findings, emphasis was placed on reproducible physiological patterns observed across independent studies.

## Results

### 1. STN-driven circuit

As previously described in a study from our group [16], the PD-related dopaminergic degeneration in PD arises from a complex interplay of mechanisms, including α-synuclein accumulation, immunometabolic dysfunction, systemic and central nervous system inflammation, glymphatic impairment and alterations in lipid-glucose metabolism. These processes ultimately lead to failure of neuronal energy homeostasis and repair mechanisms, culminating in apoptosis.

In the context of dopaminergic depletion, striatal medium spiny neurons become hyperactive and increasingly responsive to cortical oscillations in the delta–theta range (4–8 Hz) [6,17,18], particularly those expressing D2 receptors (iSPNs) [18]. This imbalance favors long-term depression and promotes functional predominance of the indirect pathway within the BG [17,18].

Increased striatal excitability leads the striatum to mirror cortical delta-theta activity in an amplified manner, effectively acting as a relay of these cortical oscillatory dynamics. This phenomenon has been demonstrated by computational simulations, experimental animal models, and neurophysiological studies in PD patients [6,10,19,20]. Accordingly, periods of heightened cortical activity correspond to states of high striatal firing (UP states), whereas periods of reduced cortical input corresponds to low-firing states (DOWN states), establishing a bistable regime [6,10,19,20,21].

These states promote an alternating pattern of striatal inhibition onto the GPe. During UP states, increased GABAergic output imposes strong inhibition on the GPe, reducing its baseline firing rate and favoring more synchronized activity [6,10,18,19,20].

This alternating inhibition results in fluctuations in GPe output to the STN, with phases of suppression alternating with disinhibition. Such dynamics have been demonstrated in biophysical models [6], computational simulations [20], and intraoperative recordings in PD patients [22], and may promote rebound bursting and sustained pathological oscillations, particularly in the delta range, consistent with tremor frequency [20].

Additionally, the sensorimotor cortex exerts a direct excitatory influence on the dorsal STN via the hyperdirect pathway [23,24], contributing to the emergence of pathological oscillations and increased network synchronization [6,25,26]. This effect appears to be more pronounced with greater dopaminergic depletion, reflecting a progressive imbalance between inhibitory and excitatory inputs to the STN, and has been associated with increased tremor severity [23,27,28,29].

During periods of STN disinhibition, its activity exerts a strong glutamatergic drive onto the GPi and, retrogradely, onto the GPe. This interaction gives rise to *state switching*, characterized by alternating phases in which the GPe inhibits STN activity, followed by phases in which the STN excites the GPe, thereby sustaining a self-organized excitatory–inhibitory oscillatory loop, as demonstrated in biophysical models and human recordings [6,24].

This heterogeneity in STN neuronal activity may contribute to the diversity of motor phenotypes observed in PD [30,31]. Within this framework, tremor-dominant symptoms may be associated with predominance of delta-theta oscillations [32], whereas rigid–akinetic symptoms may relate more closely to beta-band activity [33]. This interpretation is supported by the observation that suppression of beta activity through dopaminergic therapy or DBS improves bradykinesia and rigidity, but has less consistent effects on tremor [34].

Despite this conceptual separation, oscillatory activity within the STN can overlap across different frequency ranges [35,36]. Intraoperative single-neuron and local field potential recordings have shown that neurons exhibiting tremor-frequency activity (4–6Hz) are predominantly located in dorsal regions but may shift to low-gamma oscillations (35–55 Hz) as tremor severity changes [36]. These findings suggest that parkinsonian tremor involves dynamic transitions between oscillatory regimes within the STN [37,38,39].

Oscillatory activity generated within the STN–GPe circuit is transmitted to the GPi, particularly its ventral sensorimotor regions, where neurons exhibit activity coherent with peripheral tremor frequency (“tremor cells”) [40,41]. However, evidence also suggests that GPi neurons may contribute to the generation of pathological oscillations in the 4–6 Hz range [41]. Thus, GPi may act as an amplifier and relay of oscillatory activity, and may also contribute to its generation. This dual role results in rhythmic GABAergic output to the TH, particularly the ventral lateral (VL) region (including VIM and Vop nuclei), contributing to tremor modulation [40,41].

This inhibitory input may drive thalamic neurons into oscillatory bursting regimes, allowing them to resonate and retransmit oscillatory activity to the sensorimotor cortex, thereby closing the thalamo–cortical loop and enabling the peripheral expression of tremor [23,41,42]. Clinically, tremor frequency typically falls within the fundamental range (3–9 Hz), although higher harmonics may also be observed [42].

Importantly, the functional impact of the STN–GPe–GPi circuit appears to depend more on synchronized oscillatory activity than on absolute firing rates [43]. Pathological synchronization within this circuit likely represents a key mechanism underlying motor symptoms expression.

However, although this framework accounts for the generation and modulation of oscillatory activity, it does not fully explain the clinical phenomenology of parkinsonian tremor [33]. Tremor often responds less robustly to dopaminergic therapy than bradykinesia [44,45], and its severity may evolve independently from rigid–akinetic symptoms [46,47]. Furthermore, suppression of tremor is not always accompanied by proportional reductions in BG oscillatory activity, which may persist even in the absence of overt tremor [48]. These observations suggest that additional, partially dopamine-independent mechanisms outside the BG likely contribute to tremor generation [49].

### 2. Cerebello-thalamic circuit

The cerebello–thalamo–cortical pathway represents a major dopamine-independent component of parkinsonian tremor and is primarily integrated with BG networks through the TH, particularly the VL region [50].

Following GABAergic input from Gpi, neurons in the VL thalamus exhibit burst-firing activity after periods of hyperpolarization [51]. Microelectrode recordings have shown that these neurons can generate oscillatory activity across multiple frequency bands, including tremor-frequency oscillations (4–6 Hz), beta activity (8–30 Hz), as well as non-oscillatory firing patterns [52].

The VL thalamus maintains a close functional relationship with the cerebellum, particularly through projections from the dentate nucleus, and preferentially connects with sensorimotor cortical regions such as the supplementary motor area (SMA) and primary motor cortex (M1), thereby closing the cerebello–thalamo–cortical loop [53,54]. This circuit appears to play a critical role in tremor expression. The strength of cerebello–cortical connectivity correlates positively with tremor severity [54], surgical evidence indicates that dentate nucleus lesions can abolish tremor [55], and neuronal synchronization with peripheral tremor is more consistently observed in the VIM thalamus than within BG structures [56].

This circuit appears to play a critical role in tremor expression. The strength of cerebello–cortical connectivity correlates positively with tremor severity [54], surgical evidence indicates that dentate nucleus lesions can abolish tremor [55], and neuronal synchronization with peripheral tremor is more consistently observed in the VIM thalamus than within basal ganglia structures [56].

Multiple lines of evidenced further support the involvement of the cerebellum in tremor generation. Multiscale computational models have shown that the inclusion of cerebellar dopaminergic dysfunction is necessary to reproduce key spectral features of parkinsonism, including increased cortical and thalamic beta power, enhanced cortico–thalamic synchronization and altered Purkinje cell and deep cerebellar nuclei dynamics [57].

Additional computational studies have demonstrated that dopaminergic depletion induces synchronized tremor-band oscillations across cortex, BG and musculoskeletal system, modulating interregional coupling [58]. These findings are supported by magnetoencephalography (MEG) studies demonstrating coherent rhythmic activity across these structures at tremor frequency or its harmonics [59], as well as by neuroimaging studies showing consistent tremor-related activity within cerebellar nodes. Together, these findings suggest that the cerebellum is actively involved in the generation and maintenance of tremor-related oscillations, rather than acting solely as a passive relay [60].

Cerebellar involvement is also reflected in structural and metabolic alterations observed in PD. Magnetic resonance morphometry studies have identified subtype-specific differences in gray matter volume, including reductions in posterior cerebellar regions in the tremor-dominant subtype [61]. Similarly, FDG-PET studies have demonstrated increased metabolic activity in anterior cerebellar regions, correlating with motor symptom severity [62]. These findings have been interpreted as reflecting compensatory cerebellar mechanisms. Furthermore, cerebellar transcranial alternating current stimulation (tACS), tuned to individual tremor frequency, has been shown to reduce tremor amplitude [58], supporting the cerebellum as potential therapeutic target.

Dynamic resting-state functional connectivity studies in tremor-dominant PD have further demonstrated increased coupling between cerebellar and sensorimotor networks during specific transient states, with the strength of this interaction correlating with tremor severity [63]. This pattern suggests increased functional engagement of cerebellar circuits as tremor intensifies.

Anatomically, the cerebellum receives afferent input via the corticopontocerebellar pathway and projects predominantly through the dentate nucleus to VL thalamic nuclei [64]. Additional connections with putamen have been described in both animal models and humans studies [53,65]. Experimental evidence indicates that ventrolateral striatal lesions can reduce baseline cerebellar activity and modulate its dynamics during abnormal motor states, suggesting that cerebellar activity may respond to perturbations originating in BG circuits [65].

Within this distributed motor network, the cerebellum appears to play a role in temporal coordination and in shaping the coherence of oscillatory activity across regions. It may influence oscillatory frequency, rhythmic stability, and inter-regional synchronization, dynamically modulating emerging motor patterns. Under pathological conditions, abnormal coupling between cerebellar, cortical and BG circuits may contribute to the stabilization or amplification of tremor-related oscillations [64].

### 3. Pontine nuclei as a functional node linking the cerebellum and the BG

Beyond the TH, the pedunculopontine nucleus (PPN) may act as integrative node within motor networks. It receives afferent input from BG structures, including the substantia nigra (SN) and the STN [66,67], and sends reciprocal projections to these regions, as well as to the striatum, globus pallidus and TH [67].

Animal studies have demonstrated that stimulation of the SN can exert both inhibitory and excitatory effects on PPN neurons. In turn, PPN neurons send reciprocal glutamatergic projections back to the SN, forming a bidirectional functional circuit [66,67]. Under conditions of dopaminergic depletion, increased inhibitory input to the PPN may trigger rebound bursting activity. Together with the intrinsic pacemaker-like firing properties of PPN, neurons, this may provide a physiological substrate for the emergence of secondary oscillations [66]. Accordingly, the PPN may act as a modulator and potential amplifier of activity originating within BG circuits [66,67].

The modulatory role of the PPN is further supported by computational models in which inhibitory input from Gpi to the PPN alternates with excitatory projections from the PPN back to the Gpi, forming a GPi–PPN loop. This interaction may dynamically regulate excitability and temporal organization in both structures [68].

In addition, the PPN may influence thalamic function and contribute to the integration of BG and cerebello–thalamo-cortical circuits through projections to intralaminar, reticular, and midline thalamic nuclei, which are involved in thalamo–cortical rhythms regulation and cerebellar output pathways [67].

Within this context, it is physiologically plausible that oscillatory activity originating in the BG propagates to the PPN, which in turn may contribute to the modulation and maintenance of these rhythms, with subsequent transmission to cortical areas via the TH [67]. However, direct evidence demonstrating sustained oscillatory coherence and bidirectional causality between these structures in PD remains limited. Therefore, this interpretation should be regarded as a mechanistic hypothesis rather than an established model.

Another potential integrative pathway between BG and cerebello–thalamo-cortical circuits involves the caudal zona incerta (cZI). Low-frequency electrical stimulation of cZI during DBS procedures has been shown to induce resting tremor in humans, even in previously non-tremulous patients [69].

It has been proposed that the cZI interacts with STN and becomes increasingly responsive to slow oscillations generated within BG circuits under dopaminergic depletion [69]. From this perspective, the cZI may exert rhythmic inhibitory influences on motor thalamic nuclei, inducing periodic hyperpolarization and rebound bursting that may subsequently be transmitted to the sensorimotor cortex [69].

Anatomically, the cZI is strategically positioned along pathways receiving cerebellar afferents, enabling potential interaction between basal ganglia–derived oscillations and cerebellar inputs. Thus, the cZI may represent a functional convergence point through which pathological activity is integrated and transmitted to thalamo–cortical networks. Moreover, rather than acting solely as a relay, it may also function as a dynamic modulator of oscillatory activity, transforming BG–derived signals into patterns compatible with fundamental tremor-frequency output.

### 4. Raphe nuclei as modulators of network dynamics

Motor networks dynamics may also be modulated by the dorsal and median serotonergic raphe nuclei, as suggested by resting-state functional connectivity studies. In patients with tremor-dominant PD, connectivity between these nuclei and multiple motor-related regions, including sensorimotor cortex, TH, putamen, and cerebellum, has been shown to be reduced compared with healthy controls and other PD subtypes. Notably, this reduction correlated negatively with tremor severity [70].

These findings suggest that serotonergic systems may influence the coherence and propagation of pathological oscillations across motor networks. Accordingly, dysfunction within these pathways may contribute to the modulation of tremor expression, although causal relationships remain to be established.

## Discussion

### 1. Proposed integrative theory

Taken together, the available evidence suggests that parkinsonian tremor does not arise from a single neuronal oscillatory frequency band [71,72] or from a single isolated anatomical generator. Rather, it appears to emerge from dynamic interactions among multiple subcortical and cortical circuits [34,43,64,73,74,75].

Within this framework, we propose a conceptual hierarchical organization comprising three interdependent functional levels. The first level corresponds to the STN-driven circuit, in which electrophysiological reorganization induced by dopaminergic depletion promotes the emergence of pathological oscillations and states of rhythmic instability. This circuit may act as a major source of oscillatory perturbation within the motor network.

The second level comprises a set of modulatory structures that regulate interregional coupling, spectral coherence, and the clinical expression of tremor. These include the cerebello–thalamo–cortical loop, the cZI, PPN and the raphe nuclei. Collectively, these structures may influence the synchronization, temporal regularity, and amplitude of motor oscillations. In this context, these intermediate nodes may function as regulators of network gain and timing, determining whether latent oscillatory activity remains subclinical or become sufficiently coherent to be expressed as overt tremor.

The third level is centered primarily on the VL thalamic nuclei and their projections to the sensorimotor cortex, through which rhythmic activity is ultimately translated into peripheral muscle discharges. The thalamus likely plays a critical role as an integrative hub and resonator, combining cerebellar, BG, and cortical inputs, while also exhibiting intrinsic properties that may favor the amplification of oscillatory patterns within the tremor frequency range.

**Figure 3:**
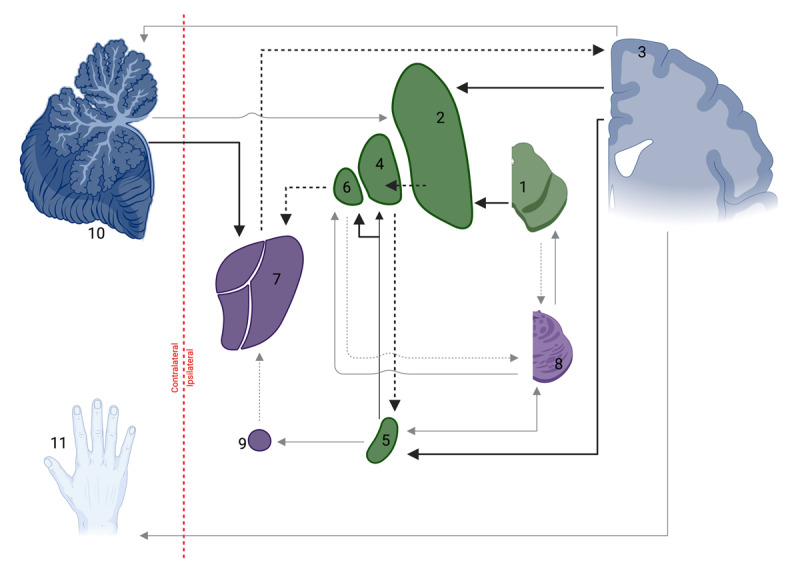
**Hierarchical organization of the distributed oscillatory network underlying parkinsonian tremor**. Schematic representation of the proposed three-level network model. Nigral dopaminergic depletion (1) leads to hyperactivity of striatal D2 medium spiny neurons (2), which become increasingly responsive to cortical oscillations (3). This state promotes an alternating pattern of striatal inhibition onto the Gpe (4), resulting in fluctuations in GPe inhibitory output to the STN (5). In parallel, the STN receives direct excitatory input from the sensorimotor cortex via the hyperdirect pathway. During periods of STN disinhibition, STN activity exerts a strong glutamatergic drive onto the GPi (6) and, retrogradely, onto the GPe. The GPi, in turn, sends rhythmic GABAergic output to the VL TH (7). The VL thalamus maintains a close functional relationship with the cerebellum (10), which receives afferent input via the corticopontocerebellar pathway and projects predominantly through the dentate nucleus to the VL thalamus and putamen. Additional modulatory structures include the PPN (8), which receives afferent input from BG structures and sends reciprocal projections to these regions and to the TH, and the cZI (9), which interacts with the STN and may exert rhythmic inhibitory influences on motor thalamic nuclei. Through these interactions, thalamic neurons resonate and retransmit oscillatory activity to the sensorimotor cortex, ultimately enabling the peripheral expression of tremor (11).

Thus, within this distributed framework, parkinsonian tremor may be understood as arising from the interaction among deep generators, subcortical modulatory nodes, and resonant thalamo–cortical circuits. This network is likely to be state-dependent and to undergo dynamic reorganization over the course of disease progression (Figure 3).

### 2. Phenotypic variation and network-level hypotheses

At present, direct evidence linking specific parkinsonian tremor phenotypes to distinct network configurations remains limited. However, converging evidence suggests that tremor heterogeneity may reflect differences in the relative contribution of dopaminergic loss, basal ganglia oscillations, cerebello–thalamo–cortical coupling, and large-scale cortical synchronization.

Rest tremor appears to involve both dopaminergic and non-dopaminergic mechanisms. Imaging studies have demonstrated an association between rest tremor severity and reduced contralateral striatal dopamine transporter binding, supporting a role for nigrostriatal degeneration in at least a subset of patients [76]. Nevertheless, the inconsistent and often incomplete response of tremor to dopaminergic therapy indicates that dopaminergic loss alone is insufficient to account for the full clinical phenotype.

Electrophysiological studies further support the role of distributed network dynamics. In particular, increased coherence between the subthalamic nucleus and cortical motor areas at tremor frequency has been directly associated with tremor manifestation and amplitude, suggesting that tremor depends on synchronized activity across multiple nodes rather than on a single subcortical generator [77]. These findings reinforce the concept of tremor as a network phenomenon involving dynamic coupling between subcortical and cortical structures.

In addition, the distinction between theta- and beta-frequency oscillations may help explain the clinical dissociation between tremor and akinetic–rigid symptoms. Tremor is consistently associated with oscillatory activity in the theta range, whereas rigidity and bradykinesia correlate more strongly with beta-band activity, suggesting partially overlapping but functionally distinct pathophysiological mechanisms [78]. This frequency-specific organization supports the hypothesis that different motor phenotypes may emerge from differential engagement of network oscillatory regimes.

Within this framework, rest tremor may be more strongly associated with intrinsic subcortical oscillatory activity and STN–cortical coupling, whereas re-emergent tremor may depend more on state-dependent reactivation of cortical and cerebello–thalamo–cortical loops during posture maintenance. Similarly, tremor-dominant phenotypes may reflect stronger cerebello–thalamo–cortical integration or a predominance of theta-range oscillatory activity, while akinetic–rigid phenotypes may be more closely linked to beta-dominant basal ganglia activity.

These interpretations remain hypothesis-generating. Direct comparative studies integrating dopaminergic imaging, invasive and non-invasive electrophysiology, tremor phenomenology, medication state, and longitudinal clinical assessment are needed to determine whether specific tremor phenotypes map onto reproducible network signatures.

### 3. Diagnostic and therapeutic implications

#### 3.1. Dopaminergic treatment

The proposed framework may help explain why dopaminergic therapy improves tremor in some patients but fails to suppress it in others. Levodopa may reduce tremor when oscillatory activity is strongly dependent on dopamine-sensitive basal ganglia dysfunction, particularly involving striatal and subthalamic nucleus (STN)–related mechanisms. However, persistence of tremor despite dopaminergic treatment may reflect the recruitment of dopamine-independent components, including cerebello–thalamo–cortical and thalamocortical circuits. Accordingly, levodopa responsiveness may not simply reflect tremor severity, but rather the relative contribution of dopamine-dependent versus dopamine-independent nodes within the network.

#### 3.2. Surgical targets and neuromodulation

From a surgical perspective, this framework helps to explain the robust clinical responses achieved with interventions targeting multiple strategic nodes within the tremor-related oscillatory network, particularly major integrative hubs such as STN, GPi, and VIM [41,79,80,81]. It also underscores the therapeutic potential of approaches aimed at modulating neuronal synchrony, including cerebellar tACs [43,58]. In addition, it broadens the scope for considering non-traditional targets, such as the cZI, whose modulation has been shown to directly influence tremor expression [82].

In parallel, several methodological advances have enabled more precise characterization of individual neurophysiological profiles, including LFP, electroencephalography (EEG), MEG and electrocorticography [35,83]. Among these, MEG has particular clinical relevance, as it allows identification of cortical sources with high spatial resolution [35]. Although these techniques are not yet widely used to guide individualized clinical decision-making, they support the notion that oscillatory patterns may serve as physiological biomarkers of distinct clinical phenotypes and symptom severity in PD.

In this context, predominance of beta-band activity, particularly within the STN, has been associated with a more favorable therapeutic response to dopaminergic therapy in the rigid-akinetic subtype [22,84]. Conversely, lower gamma-band power in the STN has been associated with greater tremor severity [34], potentially identifying patients who may benefit from more aggressive or targeted therapeutic strategies. In addition, beta–gamma coupling has been proposed as an electrophysiological marker of motor state and as a potential target for adaptive DBS strategies [85]. Similarly, resting-state cortical oscillations may enable continuous monitoring and fine-tuning of stimulation parameters [86]. Together, these findings support the use of oscillatory patterns as dynamic biomarker of therapeutic response.

Accordingly, characterization of these network dynamics may contribute to guiding the selection of therapeutic targets and strategies for each patient [31,86,87], including approaches that prioritize multi-target interventions rather than modulation of a single oscillatory generator [64]. This perspective is further supported by evidence that oscillatory patterns vary dynamically across individuals and over time, and that STN stimulation synchronized to specific phases of the tremor cycle significantly reduces tremor amplitude [88], thereby supporting the development of adaptive DBS approaches [89,90].

Finally, this framework should be interpreted as a conceptual and hypothesis-generating synthesis rather than a definitive or fully validated model.

### 4. Conceptual Advances and Novelty of the Proposed Framework

In summary, this review advances existing dual-loop and network-based models of parkinsonian tremor by proposing a fully distributed oscillatory framework in which dopaminergic depletion primarily reshapes the coupling between cortical, basal ganglia, cerebello–thalamo–cortical, and brainstem nodes rather than acting on a single pacemaker structure.

A key feature of this framework is the integration of dynamic “state-switching” interactions between the STN and Gpe as a potential mechanism underlying intermittent tremor expression. In addition, the model emphasizes the modulatory role of PPN and other mesencephalic structures in shaping basal ganglia output, and incorporates the cZI and dentate nucleus as convergent regulators of thalamic oscillatory gain.

By conceptualizing tremor as the result of context-dependent reweighting among parallel motor circuits, rather than linear propagation along isolated pathways, this framework provides a mechanistic basis for understanding tremor heterogeneity, variability across disease stages, and differential responses to DBS and pharmacological therapies.

## Limitations and open questions

Despite the integrative scope of the present framework, several limitations should be acknowledged. Much of the evidence supporting specific nodes and connections within the proposed distributed oscillatory network derives from heterogeneous sources, including small DBS cohorts, tractography-based inferences, animal models, and electrophysiological recordings obtained under varying clinical conditions.

Accordingly, the relative contribution of each pathway and their causal role in tremor generation have yet to be systematically quantified in humans. Moreover, the model does not formally account for key aspects of clinical heterogeneity, including differences between tremor-dominant and akinetic-rigid phenotypes, distinctions between resting and re-emergent tremor, and stage-dependent network reconfiguration over the course of disease progression.

Future multimodal studies integrating invasive recordings, stimulation paradigms, neuroimaging, and computational modeling will be required to test the proposed state-switching dynamics, determine whether distinct oscillatory modes correspond to specific clinical phenotypes, and identify the network nodes represent the most effective and durable therapeutic targets.

In addition, the proposed links between network dynamics and specific tremor phenotypes remain hypothetical, as direct comparative evidence is currently limited.

## Conclusions

Taken together, the available evidence suggests that parkinsonian tremor arises from dynamic interaction among multiple nodes within a distributed motor network involving the BG, TH, cerebellum, and motor cortex. The convergent contribution of these systems provides a mechanistic basis for the clinical heterogeneity of tremor, including its distinct phenotypic presentations and marked interindividual variability.

The identification of individual oscillatory signatures through LFP, EEG, and MEG opens the possibility of physiological patient stratification, more precise surgical target selection, and the development of adaptive, phase-dependent DBS strategies. Moreover, interventions aimed at modulating neural synchronization, including non-invasive cerebellar stimulation, further underscore the translational potential of multi-target and personalized therapeutic approaches.

Future investigations should integrate simultaneous multimodal recordings, causally informative stimulation paradigms, and multiscale modeling approaches to more precisely map interactions among these network structures. In addition, adaptive DBS strategies guided by physiological biomarkers, potentially combined with cerebellar modulation, represent promising avenues for optimizing tremor control. In this context, individualized characterization of network dynamics may become a central component of a precision-medicine approach to movement disorders.
